# Weighted next reaction method and parameter selection for efficient simulation of rare events in biochemical reaction systems

**DOI:** 10.1186/1687-4153-2011-797251

**Published:** 2011-07-25

**Authors:** Zhouyi Xu, Xiaodong Cai

**Affiliations:** 1 Department of Electrical and Computer Engineering, University of Miami, 1251 Memorial Drive, Coral Gables, FL 33124, USA

## Abstract

The weighted stochastic simulation algorithm (wSSA) recently developed by Kuwahara and Mura and the refined wSSA proposed by Gillespie et al. based on the importance sampling technique open the door for efficient estimation of the probability of rare events in biochemical reaction systems. In this paper, we first apply the importance sampling technique to the next reaction method (NRM) of the stochastic simulation algorithm and develop a weighted NRM (wNRM). We then develop a systematic method for selecting the values of importance sampling parameters, which can be applied to both the wSSA and the wNRM. Numerical results demonstrate that our parameter selection method can substantially improve the performance of the wSSA and the wNRM in terms of simulation efficiency and accuracy.

## 1 Introduction

Biochemical reaction systems in living cells exhibit significant stochastic fluctuations due to a small number of molecules involved in processes such as the transcription and translation of genes [1]. A number of exact [2-7] or approximate simulation algorithms [8-19] have been developed for simulating the stochastic dynamics of such systems. Recent research shows that some rare events occurring in biochemical reaction system with an extremely small probability within a specified limited time can have profound and sometimes devastating effects [20,21]. Hence, it is important that computational simulation and analysis of systems with critical rare events can efficiently capture such rare events. However, the existing exact simulation methods such as Gillespie's exact SSA [2,3] often require prohibitive computation to estimate the probability of a rare events, while the approximate methods may not be able to estimate such probability accurately.

The weighted stochastic simulation algorithm (wSSA) recently developed by Kuwahara and Mura [22] based on the importance sampling technique enables one to efficiently estimate the probability of a rare event. However, the wSSA does not provide any method for selecting optimal values for importance sampling parameters. More recently, Gillespie et al. [23] analyzed the accuracy of the results yielded from the wSSA and proposed a refined wSSA that employed a try-and-test method for selecting optimal values for importance sampling parameters. It was shown that the refined wSSA could further improve the performance of wSSA. However, the try-and-test method requires some initial guessing for the sets of values from which the parameters can take. If the guessed values do not include the optimal value, then one cannot get appropriate values for the parameters. Moreover, if the number of parameters is greater than one, a very large set of values need to be guessed and tested, which may increase the likelihood of missing the optimal values and also increase computational overhead.

In this paper, we first apply the importance sampling technique to the next reaction method (NRM) of the SSA [4] and develop a weighted NRM (wNRM) as an alternative to the wSSA. We then develop a systematic method for selecting optimal values for importance sampling parameters that can be incorporated into both the wSSA and the wNRM. Our method does not need initial guess and thus can guarantee near optimal values for the parameters. Our numerical results in Section 5 demonstrate that the variance of the estimated probability of the rare event provided by the wSSA and wNRM with our parameter selection method can be more than one order magnitude lower than that provided by the wSSA or the refined wSSA for a given number of simulation runs. Moreover, the wSSA and wNRM with our parameter selection method require less simulation time than the refined wSSA for the same number of simulation runs. When this paper was under review, a method named doubly weighted SSA (dwSSA) was developed to automatically choose parameter values for the wSSA [24]. The dwSSA reduces the computational overhead required by the wSSA and the refined wSSA to select parameter values, but it produces similar variance for the estimated probability as the refined wSSA.

The remaining part of this paper is organized as follows. In Section 2, we first describe the system setup and then briefly review Gillespie's exact SSA [2,3], the wSSA [22] and the refined wSSA [23]. In Section 3, we develop the wNRM. In Section 4, we develop a systematic method for selecting optimal values for importance sampling parameters and incorporate the parameter selection procedure into both the wSSA and the NRM. In Section 5, we give some numerical examples that illustrate the advantages of our parameter selection method. Finally in Section 6, we draw several conclusions.

## 2 Weighted stochastic simulation algorithms

### 2.1 System Description

Suppose a chemical reaction system involves a well-stirred mixture of *N *≥ 1 molecular species {*S*_1_, ..., *S_N_*} that chemically interact through *M *≥ 1 reaction channels {*R*_1_, ..., *R_M_*}. The dynamic state of this chemical system is described by the state vector **X**(*t*) = [*X*_1_(*t*), ..., *X_N_*(*t*)]*^T^*, where *X_n_*(*t*), *n *= 1, ..., *N*, is the number of *S_n _*molecules at time *t*, and [·]*^T ^*denotes the transpose of the vector in the brackets. Following Gillespie [8], we define the dynamics of reaction *R_m _*by a state-change vector ***ν***_*m *_= [*ν*_1*m*_, ..., *ν_Nm_*]*^T^*, where *ν_nm _*gives the changes in the *S_n _*molecular population produced by one *R_m _*reaction, and a propensity function *a_m_*(**x**) together with the fundamental premise of stochastic chemical kinetics:(1)

### 2.2 Gillespie's exact SSA

Based on the fundamental premise (1), Gillespie developed an exact SSA to simulate the occurrence of every reaction when the time evolves [3]. Specifically, Gillespie's SSA simulates the following event in each step:(2)

It has been shown by Gillespie [2,3] that *τ *and *μ *are two independent random variables and have the following probability density functions (PDF) and probability mass function (PMF), respectively,(3)

and(4)

where . Therefore, Gillespie's direct method (DM) for the SSA generates a realization of *τ *and *μ *according to PDF (3) and PMF (4), respectively, in each step of the simulation, and then updates the system state as **X**(*t *+ *τ*) = **x **+ ***ν***_*μ*_.

### 2.3 Weighted SSA

In order to estimate the probability of a rare event that occurs with an extremely low probability in a given time period, Gillespie's SSA may require huge computation. Recently, the wSSA [22] and the refined wSSA [23] were developed to estimate the probability of a rare event with substantial reduction of computation. Following Kuwahara and Mura [22], and Gillespie et al. [23], we define the rare event *E_R _*as follows:(5)

If we employ the SSA to estimate *P*(*E_R_*), we would have to make a large number *n *of simulation runs, with each starting at time 0 in state **x**_0 _and terminating either when some state **x **∈ Ω is first reached or when the system time reaches *T*. If *k *is the number of those *n *runs that terminate for the first reason, then *P*(*E_R_*) is estimated as . Since *P*(*E_R_*) ≪ 1, *n *should be very large to get a reasonably accurate estimate of *P*(*E_R_*). The wSSA employs the importance sampling technique to reduce the number of runs needed to estimate *P*(*E_R_*).

Specifically, wSSA generates *τ *from its PDF (3) in the same way as used in Gillespie's DM method, but generates the reaction index *μ *from the following PMF:(6)

where *b_μ_*(**x**) = *γ_μ _a_μ_*(**x**), *μ *= 1, ..., *M*,  and *γ_μ_*, *μ *= 1, ..., *M *are positive constants that need to be chosen carefully before simulations are run. Suppose a trajectory *J *generated in a simulation run contains *h *reactions and the *i*th reaction occurs at time *t_i_*, then the wSSA changes the PDF of the trajectory from  to , where *t*_0 _= 0. By choosing appropriate *γ_μ_*, *μ *= 1, ..., *M*, one can increase the probability of the trajectories that lead to the rare event. If *k *trajectories out of *n *simulation runs lead to the rare event, then the importance sampling technique tells us that an unbiased estimate of *P*(*E_R_*) is given by(7)

where *j *and *i *are indices of the trajectories and reactions in a trajectory, respectively, , and(8)

which can be obtained in each simulation step.

Kuwahara and Mura [22] did not provide any method for choosing *γ_μ_*, although their numerical results with some pre-specified *γ_μ _*for several reaction systems demonstrated that the wSSA could reduce computation substantially. Gillespie et al. [23] analyzed the variance of  obtained from the wSSA and refined the wSSA by proposing a try-and-test method for choosing *γ_μ_*. In the try-and-test method, several sets of values are pre-specified for *γ_μ_*, *μ *= 1, ..., *M*. A relatively small number of simulation runs of the standard SSA are made for each set of the values to obtain an estimate of the variance of , and then the set of values that yielded the smallest variance is chosen. Although the try-and-test method provides a way of choosing *γ_μ_*, it requires some guessing to get several sets of pre-specified values for all *γ_μ _*and also some computational overhead to estimate the variance of  for each set of values. More recently, the dwSSA was developed in [24] to automatically choose parameter values for the wSSA by applying the cross-entropy method originally proposed in [25] for optimizing the importance sampling method.

## 3 Weighted NRM

The wSSA is based on the DM for the SSA, which needs to generate two random variables in each simulation step. However, the NRM of Gibson and Bruck [4] requires only one random variable in each simulation step. In this section, we apply the importance sampling technique to the NRM and develop the wNRM.

The key to making the wSSA more efficient than the standard SSA is to change the probability of each reaction appropriately but without changing the distribution of the time *τ *between any two consecutive reactions. Since the NRM determines the reaction occurring in a simulation step by choosing the reaction that requires the smallest waiting time, it seems difficult to change the probability of each reaction without changing the distribution of *τ*. However, we notice that the PDF of *τ *in (3) only depends on *a*_0_(**x**) not individual *a_μ_*(**x**). Hence, we can change the probability of each reaction by changing the corresponding propensity function but without changing the distribution of *τ*, so long as we keep the sum of the propensity functions equal to *a*_0_(**x**). To this end, we define(9)

where *b_m_*(**x**) = *γ_m_a_m_*(**x**) is defined in the same way as in the wSSA. It is easy to verify that . If we generate *τ_m _*from an exponential distribution *p*(*τ_m_*) = *d_m_*(**x**) exp(-*d_m_*(**x**)*τ_m_*), *τ_m _*> 0, as the waiting time of reaction channel *m*, and choose *μ *= arg*_m _*min{*τ_m_*, *m *= 1, ..., *M*} as the index of the channel that fires, then it can be easily shown that the PDF of *τ *= min{*τ_m_*, *m *= 1, ..., *M*} follows the exponential distribution in (3) and that the probability of reaction *μ *is *q_μ _*= *d_μ_*(**x**)/*d*_0_(**x**) = *b_μ_*(**x**)/*b*_0_(**x**). If we repeat this procedure in each simulation step, we would have modified the first reaction method (FRM) [3] for the standard SSA and got a weighted FRM (wFRM). Clearly, the wFRM is not efficient since it generates *M *random variables in each step. However, following Gibson and Bruck [4], we can convert the wFRM into a more efficient wNRM by reusing *τ_m_*s.

In the FRM, we used *τ_m _*to denote the putative waiting or relative time for the *m*th reaction channel to fire. Following Gibson and Bruck [4], we will use *τ_m _*to denote the putative absolute time when the *m*th reaction channel will fire. Suppose that the *μ*th reaction channel fires at time *t *in the current step. After updating the state vector and propensity functions, we calculate new *d_m_*(**x**), *m *= 1, ..., *M*, which we denote as . Then, we generate a random variable  from an exponential distribution with parameter  and set . For other channels with an index *m *≠ *μ*, we update *τ_m _*as follows:(10)

Following Gibson and Bruck [4], we can show that the new *τ_m _*-*t*, *m *= 1, ..., *M*, are independent exponential random variables with parameters , *m *= 1, ..., *M*, respectively. Therefore, in the next step, we can choose *μ *= arg*_m _*min{*τ_m_*, *m *= 1, ..., *M*} as the index of the channel that fires as done in NRM, update *t *as *t *= *τ_μ_*, and then repeat the process just described. Clearly, the wNRM only needs to generate one random variable in each step. We can further improve the efficiency of the wNRM by using the dependency graph  and the indexed priority queue  defined by Gibson and Bruck [4]. The dependency graph  tells precisely which propensity functions need to be updated after a reaction occurs. The indexed priority queue  can be exploited to find the minimum *τ_m _*and the reaction index in each step more efficiently than finding the reaction index from the PMF (4) directly as done in the DM. However, some computational overhead is needed to maintain the data structure of .

Essentially, our wNRM runs simulation in the same way as the NRM except that the wNM generates *τ_m _*using a parameter *d_m_*(**x**) instead of *a_m_*(**x**). To estimate the probability of the rare event , we calculate a weight  in each step and get  using (7). The wNRM is summarized in the following algorithm:

Algorithm 1 (wNRM)

*1. k*_1 _← 0*, k*_2 _← 0*, set values for all γ_m_; generate a dependency graph *.

*2*. **for ***i = 1 to n*, **do**

*3.   t *← 0, **x **← **x**_0_*, w *← 1.

*4.   evaluate a_m_*(**x**) *and b_m_*(**x**) *for all m; calculate all d_m_*(**x**).

*5.   for each m, generate a unit-interval uniform random variable r_m_; τ_m _*= ln(1/*r_m_*)/*d_m_*(**x**).

*6.   store τ_m _in an indexed priority queue *.

*7.*   **while ***t *≤ *T*, **do**

*8.*      **if x **∈ Ω, **then**

*9.         k*_1 _← *k*_1 _+ *w, k*_2 _← *k*_2 _+ *w*^2^

10.         break out the while loop

*11.*      **end if**

*12.      find μ *= arg*_m _*min{*τ_m_*, *m *= 1, ..., *M*} *and τ *= min{*τ_m_*, *m *= 1, ..., *M*} *from *.

*13.      w *← *w *× *a_μ_*(**x**)*/d_μ_*(**x**).

*14.*      **x **← **x **+ ***ν***_*μ*_, *t *← *τ*.

*15.      Find a_m_*(**x**) *need to be updated from **; evaluate these a_m_*(**x**) *and the corresponding b_m_*(**x**)*; calculate all *.

*16.      for all m *≠ *μ*, *; generate a unit-interval uniform random variable r*_*μ*_; *; update *.

*17.*      .

*18.*   **end while**

*19. ***end for**

*20. *

*21. calculate **, with a 68% uncertainty of *.

Note that Gibson and Bruck [4] argued that the NRM is more efficient than the DM of Gillespie's SSA for the loosely coupled chemical reaction systems. On the other hand, Cao et al. [5] optimized the DM and argued that the optimized DM is more efficient for most practical reaction systems. Regardless of the debate about the efficiency, here we propose the wNRM as an alternative of the wSSA which is based on the DM. While our simulation results in Section 5 demonstrate that the wNRM is more efficient than the refined wSSA for the three reaction systems tested, the wSSA may be more efficient in simulating some other systems.

As in the wSSA, Algorithm 1 does not provide a method for selecting the values of parameters *γ_m_*, *m *= 1, ..., *M*. Although we could incorporate the try-and-test method in refined wSSA into Algorithm 1, we will develop a more systematic method for selecting parameters in the next section. This parameter selection method will be applicable to both the wSSA and the wNRM and can significantly improve the performance of both algorithms as will be demonstrated in Section 5.

## 4 Parameter selection for wSSA and wNRM

Let us denote the set of all possible state trajectories in the time interval [0 *T*] as  and the set of trajectories that first reach any state in Ω during [0 *T*] as . Let the probability of a trajectory *J *be *P_J_*. Then, we have , where the indicator function  if  or 0 if . Importance sampling used in the wSSA and the wNRM arises from the factor that we can write *P*(*E_R_*) as(11)

where *Q_J _*is the probability used in simulation to generate trajectory *J*, which is different from the true probability *P_J _*if the original system evolves naturally. If we make *n *simulation runs with altered trajectory probabilities, (11) implies that we can estimate *P*(*E_R_*) as  which is essentially (7). The variance of  depends on *Q_J_*s. Appropriate *Q_J_*s yield small variance, thereby improving the accuracy of the estimate or equivalently reducing the number of runs for a given variance. The "rule of thumb" [23,26-28] for choosing good *Q_J_*s is that *Q_J _*should be roughly proportional to . However, at least two difficulties arise if we apply the rule of thumb based on (11). First, the number of all possible trajectories is very large and we do not know the trajectories that lead to the rare event and their probabilities. Second, since we can only adjust the probability of each reaction in each step, it is not clear how this adjustment can affect the probability of a trajectory. To overcome these difficulties, we next use an alternative expression for *P*(*E_R_*) based on which we apply the importance sampling technique.

Let us denote the number of reactions occurring in the time interval [0 *t*] as *K_t _*and the maximum value of *K_T _*as . Let *E_K _*be the rare event occurring at the *K*th  reaction at any *t *≤ *T*, and *P*(*E_K_*) be the probability of *E_K _*in the original system that evolve naturally with the original probability rate constants. Then, we have(12)

If *Q*(*E_K_*) is the probability of event *E_K _*in the weighted system that evolves with adjusted probability rate constants, the rule of thumb for choosing good *Q*(*E_K_*) is that we should make *Q*(*E_K_*) approximately proportional to *P*(*E_K_*). However, it is still difficult to apply the rule of thumb, because it is difficult to control every *Q*(*E_K_*) simultaneously. Hence, we relax the rule of thumb and will maximize the *Q*(*E_K_*) corresponding to the maximum *P*(*E_K_*) or the one near maximum if the exact maximum *P*(*E_K_*) cannot be determined precisely. The rationale of this heuristic rule is based on the following argument. If  is the maximum one among all , the sum of  and its closely related terms, such as , ,  and , very likely dominates the sum in the right-hand side of (12). Maximizing  not only proportionally increases , and its closely related terms, such as , ,  and , but also significantly increases the probability of the occurrence of the rare event. Note that a similar heuristic rule relying on the event with maximum probability was proposed in [29] for estimating the probability of rare events in highly reliable Markovian systems.

Before proceeding with our derivations, we need to specify Ω. In the rest of the paper, we assume that Ω contains one single state **X **defined as *X_i _*= *X_i_*(0) + *η*, where *η *is a constant and *i *∈ {1, 2, ..., *N*}. Let us denote the number of firings of the *m*th reaction channel in the trajectory leading to the rare event as *K_m_*. Then, we have(13)

We first divide all reactions into three groups using the following general rule: *G*_1 _group consists of reactions with *ν_im_η *> 0, *G*_2 _group consists of reactions with *ν_im_η *< 0, and *G*_3 _group consists of reactions with *ν_im _*= 0. The rationale for the partition rule is that the reactions in *G*_1 _(*G*_2_) group increase (decrease) the probability of the rare event and that the reactions in *G*_3 _group do not affect *X_i_*(*t*) directly. We further refine the partition rule as follows. If a reaction *R_m _*is in the *G*_1 _group based on the general rule but *a_m_*(**x**) = 0 whenever one *R_m _*reaction occurs, we move *R_m _*into the *G*_3 _group. Similarly, if a reaction *R_m _*is in the *G*_2 _group based on the general rule but *a_m_*(**x**) = 0 whenever one *R_m _*reaction occurs, we move *R_m _*into the *G*_3 _group. For most cases, we only need the general partition rule. The refining rule described here is to deal with the situation where one or several *X_i_*(*t*)s always take values 1 or 0 as in the system considered in Section 5.3. More refining rules may be added following the rationale just described, after we see more real-world reaction systems.

We typically only need to consider elementary reactions including bimolecular and monomolecular reactions [30]. Hence, the possible values for all *ν_im _*are 0, ±1, ±2. For the simplicity of derivations, we now only consider the case where *ν_im _*= 0, ±1, i.e., we assume that the system does not have any bimolecular reactions with two identical reactant molecules or dimerization reactions. We will later generalize our method to the system with dimerization reactions. Let us define ,  and , then (13) becomes(14)

Let us denote  as the expected value of *K_t_*. Since the number of reactions occurring in any small time interval is approximately a Poisson random variable [8], *K_t _*is the sum of a large number of independent Poisson random variables when *t *is relatively large. Then, by the central limit theorem, *K_t _*can be approximated as a Gaussian random variable with mean . Indeed, in all chemical reaction systems [6,19,31] we tested so far, we observed that *Kt *follows a unimodal distribution with a peak at  and its standard deviation is small relative to . Since the mean first passage time of the rare event is much larger than *T *[23], the rare event most likely occurs at a time near *T*. Based on these two observations, we argue that  for all . Therefore, we should have . When  occurs, we have(15)

Since both (14) and (15) need to be satisfied in order for the event  to occur and since ,  and , we get the second requirement for *K_E_*: *K_E _*≥ |*η*|. Combining the two requirements on *K_E_*, we obtain .

The probability *P*(*E_K_*) can be expressed as . Since *P*(**X**(*t*) ∈ Ω|*K_t _*= *K*) is determined by the constant *K*, it is independent of *t*. Hence, we have . Due to the unimodal distribution of *K_t _*we mentioned earlier, we have  for those ;  for those *K *close to ; and  quickly decreases to zero when *K *increases beyond . In other words,  is approximately a constant for  and quickly decreases to zero when . Now let us consider event *E_K _*with *K *= |*η*| in the case . In this case, *P *(**X**(*t*) ∈ Ω|*K_t _*= *K*) is very small because this is an extreme case where  and  if *η *> 0 or  and  if *η *< 0. Therefore, we can increase *P*(*E_K_*) if we increase *K*, but we do not want to increase *K *too much because as we discussed  decreases quickly when *K *increases in the case . Consequently, we suggest that we choose , where  is the standard deviation of *K_T _*which can be estimated by making hundreds of runs of the standard SSA. In case , we choose  based on the same argument that  decreases quickly if we further increase *K_E_*.

Applying the relaxed rule of thumb, we need to adjust probability rate constants in simulation to maximize . Since we do not change the distribution of *τ*, we do not change the distribution of *K_T _*and thus . Hence, maximizing *Q*(*E_K_*) is equivalent to maximizing *Q*(**X**(*t*) ∈ Ω|*K_t _*= *K_E_*). Now we are in a position to summarize our strategy of applying the important sampling technique in simulation as follows: we will choose probability parameters to maximize *Q*(**X**(*t*) ∈ Ω|*K_t _*= *K_E_*), where(16)

We next consider systems with only *G*_1 _and *G*_2 _reaction groups and then consider more general systems with all three reaction groups.

### 4.1 Systems with *G*_1 _and *G*_2 _reaction groups

Since we do not have *G*_3 _group, (15) becomes(17)

Combining (14) and (17), we get  and  if the final state after the last reaction occurs is in Ω. The last reaction should be a reaction from *G*_1 _group. Otherwise, the state already reached Ω before the last reaction occurs. Suppose that in simulation the total probability of the occurrence of reactions in *G*_1 _group is a constant  and then the total probability of the occurrence of reactions in *G*_2 _group is . Then, *Q*(**X**(*t*) ∈ Ω|*K_t _*= *K_E_*) can be found from a binomial distribution as follows(18)

where  and  as determined earlier. Setting the derivative of *Q*(**X**(*t*) ∈ Ω|*K_t _*= *K_E_*) with respect to  to be zero, we get  and  that maximize *Q*(**X**(*t*) ∈ Ω|*K_t _*= *K_E_*) as follows:(19)

To ensure that reactions in *G*_1 _(*G*_2_) group occur with probability  in each step of simulation, we adjust the probability of each reaction as follows(20)

where  and . It is easy to verify that  and . As defined in (8), the weight for estimating the probability of the rare event is *w_μ _*= *p_μ_*/*q_μ _*if the *μ*th reaction channel fires.

### 4.2 Systems with *G*_1_, *G*_2 _and *G*_3 _reaction groups

Combining (14) and (15), we get  and . Since , we have . Suppose that in simulation the total probabilities of the occurrence of reactions in *G*_1_, *G*_2 _and *G*_3 _are constants ,  and , respectively. Then, *Q*(**X**(*t*) ∈ Ω|*K_t _*= *K_E_*) can be found from a multinomial distribution as follows(21)

where  and  as determined earlier. Since there are (*K_E _- η*)/2 + 1 terms of the sum in (21), it is difficult to find ,  and  that maximize *Q*(**X**(*t*) ∈ Ω|*K_t _*= *K_E_*). So we will use a different approach to find ,  and  as described in the following.

Let ,  and  be the average number of reactions of *G*_1_, *G*_2 _and *G*_3 _groups that occur in the time interval [0 *T*] in the original system. Since we have , we define , , and . Then, we can approximate *P*(**X**(*t*) ∈ Ω|*K_t _*= *K_E_*) in the original system, which is the counter part of *Q*(**X**(*t*) ∈ Ω|*K_t _*= *K_E_*) in the weighted system, using the right-hand side of (21) but with , *i *= 1, 2, 3, replaced by *i *= 1, 2, 3, respectively. This gives(22)

Suppose that the (*κ *+ 1)th term of the sum in (22) is the largest. We further relax the rule of thumb and maximize the (*κ *+ 1)th term of the sum in (21) to find ,  and .

It is not difficult to find the (*κ *+ 1)th term of the sum in (22). Let us denote the  term of the sum in (22) as . We can exhaustively search over all ,  to find *κ*. However, this may require relatively large computation because the factorials involved in . We can reduce computation by searching over , , which are given by(23)

Specifically, we calculate all  from (23). If  but , then  is a local maximum. After obtaining all local maximums, we can find the global maximum *f*(*κ*) from the local maximums.

After we find *κ*, we set the partial derivatives of the (*κ *+ 1)th term of the sum in (21) with respect to  and  to be zero. This gives the following optimal ,  and (24)

Substituting  and  in (24) into (20), we get the probability *q_m_*, *m *∈ *G*_1 _or *G*_2 _that is used to generate the *m*th reaction in each step of simulation. For *G*_3 _group, we get the probability of each reaction as follows(25)

where .

While we can use *q_m _*in (25) to generate reactions in *G*_3 _group, we next develop an optional method for fine-tuning *q_m_*, *m *∈ *G*_3_, which can further reduce the variance of . We divide *G*_3 _group into three subgroups: *G*_31_, *G*_32 _and *G*_33_. Occurrence of reactions in *G*_31 _group increases the probability of occurrence of reactions in  group or reduces the probability of the occurrence of the reactions in  group, which in turn increases the probability of the rare event. Occurrence of reactions in *G*_32 _group reduces the probability of occurrence of reactions in  group or increases he probability of the occurrence of reactions in  group, which reduces the probability of the are event. Occurrence of reactions in *G*_33 _group does not change the probability of occurrence of reactions in  and  groups, which does not change the probability of the rare event.

Let ,  and  be the average number of reactions from *G*_31_, *G*_32 _and *G*_33 _that occur in the time interval [0 *T*] in the original system. we define ,  and . Our goal is to make *Q*_31 _to be greater than  and *Q*_32 _to be less than  to increase the probability of the rare event. However, this may not feasible when . Hence, we can fine-tune ,  and  only when  and propose the following formula to determine *Q*_31_, *Q*_32 _and *Q*_33_:(26)

where *α*, *β *∈ (0 1) are two pre-specified constants. It is not difficult to verify from (26) that . To ensure that  and , we choose *α *and *β *satisfying 0 ≤ *β *< 1 and .

Finally, we obtain *q_m _*for *m *∈ *G*_3 _as follows(27)

where , *i *= 1, 2, 3.

### 4.3 Systems with dimerization reactions

So far we assumed that the system did not have any dimerization reactions, i.e. the system consisted of reactions with |*ν_im_*| = 0 or 1. We now generalize our methods developed earlier to the system with dimerization reactions. If there are dimerization reactions in *G*_1 _and *G*_2 _groups, we further divide *G*_1 _group into *G*_11 _and *G*_12 _subgroups and *G*_2 _group into *G*_21 _and *G*_22 _subgroups. The *G*_11 _group contains reactions with *ν_im_*sign(*η*) = 1, where sign(*η*) = 1 when *η *> 0 and sign(*η*) = -1 when *η *< 0. The *G*_12 _group contains reactions with *ν_im_*sign(*η*) = 2. The *G*_21 _group contains reactions with *ν_im_*sign(*η*) = -1, while the *G*_12 _group contains reactions with *ν_im_*sign(*η*) = -2.

Let us define , ,  and . Clearly, we have  and . Then, (13) becomes(28)

Let us consider systems with *G*_1 _and *G*_2 _groups but without *G*_3 _group. Although we still have  or equivalently , we cannot obtain four unknowns , ,  and  from only two equations.

Suppose that , ,  and  are average number of reactions from *G*_11_, *G*_12_, *G*_21 _and *G*_22 _groups that occur in the time interval [0 *T*] in the original system. We notice from (20) that we do not change the ratio of the probabilities of two reactions in the same group, i.e.,  if *m*_1 _and *m*_2 _are in the same *G*_1 _or *G*_2 _group. Therefore, we would expect that  and . Using these two relationships, we can write (28) as(29)

where  and .

From (17) and (29), we obtain  and . Substituting  and  into (18) and maximizing *Q*(**X**(*t*) ∈ Ω|*K_t _*= *K_E_*), we obtain(30)

We then substitute  and  into (20) to get *q_m_*.

Now let us consider the systems with *G*_1_, *G*_2 _and *G*_3 _reactions. From (29), we have , and from (15) and (29), we obtain . Since , we have . Following the derivations in Section 4.2, we can get *q_m _*for any reaction. More specifically, substituting ,  and the upper limit of  into (21), we obtain *Q*(**X**(*t*) ∈ Ω|*K_t _*= *K_E_*). We can also get *P*(**X**(*t*) ∈ Ω|*K_t _*= *K_E_*) similar to (22) by replacing  in *Q*(**X**(*t*) ∈ Ω|*K_t _*= *K_E_*) with . Then, we determine the maximum term of the sum in *P*(**X**(*t*) ∈ Ω|*K_t _*= *K_E_*) and denote the value of  corresponding to the maximum term as *κ *+ 1. We find ,  and  by maximizing the (*κ *+ 1)th term of the sum in *Q*(**X**(*t*) ∈ Ω|*K_t _*= *K_E_*). Finally, we substitute  and  into (20) to get *q_m_*, *m *∈ *G*_1 _or *G*_2_. For the reactions in *G*_3 _group, we can either substitute  into (25) to obtain *q_m_*, or if we want to fine-tune *q_m_*, we use (26) and (27) to get *q_m_*.

### 4.4 wSSA and wNRM with parameter selection

The key to determining probability of each reaction *q_m _*is to find the total probability of each group, , , , ,  and . This requires the average number of reactions of each group occurring during the interval [0 *T*] in the original system, , , , , , , , . If the system is relatively simple, we may get these numbers analytically. If we cannot obtain them analytically, we can estimate them by running Gillespie's exact SSA. Since the number of runs needed to estimates these numbers is much smaller than the number of runs needed to estimate the probability of the rare event, the computational overhead is negligible.

We next summarize the wSSA incorporating the parameter selection method in the following algorithm. We will not include the procedure for fine-tuning the probability rate constants of reactions in the *G*_3 _group, but will describe how to add this optional procedure to the algorithm. We will also describe how to modify Algorithm 1 to incorporate the parameter selection procedure into the wNRM.

Algorithm 2 (wSSA with parameter selection)

*1. run Gillespie's exact SSA *10^3^-10^4 ^*times to get estimates of *, , , , , *and *; *determine K_E _from *(16).

*2. if the system has only G*_1 _*and G*_2 _*reactions, calculate **and **from *(19) *if there is no dimerization reaction or from *(30) *if there are dimerization reaction(s)*, *if the system has G*_1_*, G*_2 _*and G*_3 _*reactions, calculate *, *and **from *(24).

*3. k*_1 _← 0*, k*_2 _← 0.

*4. ***for ***i *= 1 *to n*, **do**

*5.   t *← 0, **x **← **x**_0_*, w *← 1.

*6.*   **while ***t *≤ *T*, **do**

*7.*      **if x **∈ Ω, **then**

*8.         k*_1 _← *k*_1 _+ *w, k*_2 _← *k*_2 _+ *w*^2^

9.         break out the while loop

*10.*      **end if**

*11.      evaluate all a_m_*(**x**)*; calculate a*_0_(**x**).

*12.      generate two unit-interval uniform random variables r*_1 _*and r*_2_.

*13.      τ *← ln(1/*r*)1)/*a*_0_(**x**)

*14.      calculate all q_m _from *(20) *and *(25).

*15.      μ *← *smallest integer satisfying *.

*16.      w *← *w *× (*a_μ_*(**x**)/*a*_0_(**x**))/(*q_μ_*(**x**)/*q*_0_(**x**)).

*17.*      **x **← **x **+ ***ν***_*μ*_, *t *← *t *+ *τ*.

*18.*   **end while**

*19. ***end for**

*20. *

*21. estimate **, with a 68% uncertainty of *.

If  and we want to fine-tune the probability rate constants of the reactions in the *G*_3 _group, we modify Algorithm 2 as follows. In step 1, we also estimate ,  and  and choose the value of *α *and *β *in (26). In step 2, we also calculate ,  and  from (26). In step 14, we calculate *q_m _*for *G*_3 _reactions from (27) instead of (25). Comparing with the refined wSSA [23], the wSSA with our parameter selection procedure does not need to make some guessing about the parameters for adjusting the probability of each reaction *q_m_*, but directly calculate *q_m _*using a systematically developed method. This has two main advantages. First, our method will always adjust *q_m _*appropriately to reduce the variance of , whereas the refined wSSA may not adjust *q_m _*as well as our method, especially if the initial guessed values are far away from the optimal values. Second, as we mentioned earlier, the computational overhead of our method is negligible, whereas the refined wSSA requires non-negligible computational overhead for determining parameters. Indeed, as we will show in Section 5, the variance of  provided by the wSSA with our parameter selection method can be more than one order of magnitude lower than that provided by the refined wSSA for given number of *n*. Moreover, the wSSA with our parameter selection method is faster than the refined wSSA, since it requires less computational overhead to adjust *q_m_*.

We can also incorporate our parameter selection method without the fine-tuning procedure into the wNRM as follows. We replace the first step of Algorithm 1 with the first three steps of Algorithm 2. We then modify the fourth step of Algorithm 1 as follows: evaluate all *a_m_*(**x**), calculate all *q_m _*from (20) and (25), and calculate all *d_m_*(**x**) as *d_m_*(**x**) = *q_m_a*_0_(**x**). Finally, we change the fifth step of Algorithm 1 to the following: find *a_m_*(**x**) need to be updated from  and evaluate these *a_m_*(**x**); calculate all *q_m _*from (20) and (25), and calculate all  as . We can also fine-tune the probability rate constants of *G*_3 _reactions in the wNRM in the same way as described in the previous paragraph for the wSSA. Note that since our parameter selection method employs a systematic method for partitioning reactions into three groups as discussed earlier, our method can be applied to any real chemical reaction systems.

## 5 Numerical examples

In this section, we present simulation results for several chemical reaction systems to demonstrate the accuracy and efficiency of the wSSA and wNRM with our parameter selection method, which we refer to as wSSAps and wNRMps, respectively, in the rest of the paper. All simulations were run in Matlab on a PC with an Intel dual Core 2.67-GHz CPU and 3G-byte memory running Windows XP.

### 5.1 Single species production-degradation model

This simple system was originally used by Kuwahara and Mura [22] and then Gillespie et al. [23] to test the wSSA and the refined wSSA. It includes the following two chemical reactions:(31)

In reaction *R*_1_, species *S*_1 _synthesizes species *S*_2 _with a probability rate constant *c*_1_, while in reaction *R*_2_, species *S*_2 _is degraded with a probability rate constant *c*_2_. We used the same initial state and probability rate constants as used in [22,23]: *X*_1_(0) = 1, *X*_2_(0) = 40, *c*_1 _= 1 and *c*_2 _= 0.025.

It is observed that the system is at equilibrium, since *a*_1_(**x**_0_) = *c*_1 _× *X*_1_(0) = *c*_2 _× *X*_2_(0) = *a*_2_(**x**_0_). It can be shown [22] that *X*_2_(*t*) is a Poisson random variable with mean equal to 40. References [22,23] sought to estimate *P*(*E_R_*) = *P*_*t*≤100_(*X*_2 _→ *θ*|**x**_0_), the probability of *X*_2_(*t*) = *θ *for *t *≤ 100 and several values of *θ *between 65 and 80. Since *θ *is about four to six standard deviations above the mean value 40, *P*_*t*≤100_(*X*_2 _→ *θ*|**x**_0_) is very small.

Kuwahara and Mura [22] employed the wSSA to estimate *P*(*E_R_*) and used *b*_1_(**x**) = *δa*_1_(**x**) and *b*_2_(**x**) = 1/*δa*_2_(**x**) with *δ *= 1.2 for four different values of *θ*: 65, 70, 75 and 80. Gillespie et al. [23] applied the refined wSSA to estimate *P*(*ER*) and used the same way to determine *b*_1_(**x**) and *b*_2_(**x**) but found that *δ *= 1.2 is near optimal for *θ *= 65 and that *δ *= 1.3 is near optimal for *θ *= 80. We repeated the simulation of Gillespie et al. [23] for *θ *= 65, 70, 75 and 80 with *δ *= 1.2, 1.25, 1.25 and 1.3, respectively. We then applied the wSSAps and the wNRMps to estimate *P*(*E_R_*) for *θ *= 65, 70, 75 and 80. This system has only two types of reaction: *R*_1 _is a *G*_1 _reaction and *R*_2 _is a *G*_2 _reaction. Since the system is at equilibrium with *a*_0_(**x**_0_) = 2,  with *T *= 100 is estimated to be 200, and thus . Using (19), we get  and *q*_2 _= 1 - *q*_1_.

**Figure 1:**
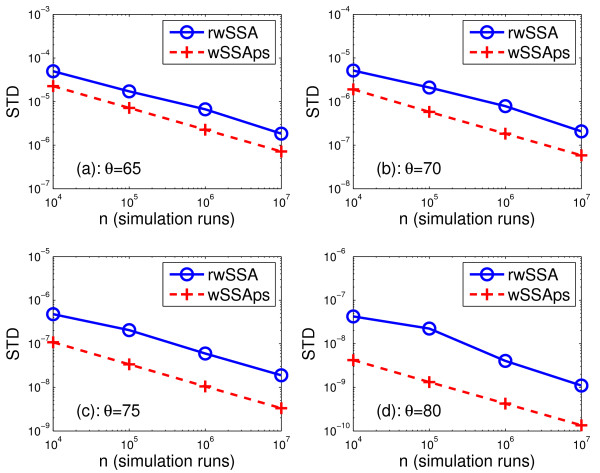
**The standard deviation (SD)  of the estimated probability versus the number of simulation runs *n *obtained with the refined wSSA (rwSSA) and the wSSAps for the single species production-degradation model (31) with *c*_1 _= 1, *c*_2 _= 0.025, *X*_1_(0) = 1 and *X*_2_(0) = 40 for *θ *= 65, 70, 75 and 80**.

Table 1 gives the estimated probability  and the sample variance *σ*^2 ^for the wNRMps, the wSSAps and the refined wSSA, obtained from 10^7 ^simulation runs with *θ *= 65, 70, 75 and 80. It is seen that  is almost identical for all three methods. However, the wNRMps and the wSSAps provide variance almost two order of magnitude lower than the refined wSSA for *θ *= 80, or less than or almost one order of magnitude lower than the refined wSSA for *θ *= 75, 70 and 65. Moreover, the wNRMps and the wSSAps need about 60 and 70% CPU time of the refined wSSA, respectively. Note that the CPU time for the refined wSSA in Table 1 does not include the time needed for searching for the optimal value of *δ *for each *θ*. The less CPU time used by the wNRMps is expected since it only requires to generate one random variable in each step, whereas the wSSAps and the refined wSSA need to generate two random variables. It is also reasonable that the wSSAps requires less CPU time than the refined wSSA, because the wSSAps needs less computation to calculate the probability of each reaction in each step. Figure 1 compares the standard deviation  of  for the wSSAps and the refined wSSA with different number of runs, *n*. Since the wNRMps provides almost the same standard deviation as the wSSAps, we do not plot it in the figure. It is seen that the wSSAps consistently yields much smaller standard deviation than the refined wSSA for all values of *n*. It was shown in [24] that the dwSSA yielded similar variance comparing to the refined wSSA. Therefore, our parameter selection method also substantially outperforms the dwSSA in this example.

**Table 1:** Estimated probability of the rare event  and the sample variance *σ*^2 ^as well as the CPU time (in s) with 10^7 ^runs of the wNRMps, the wSSAps and the refined wSSA for the single species production-degradation model (31): (a) *θ *= 65 and 70 and (b) *θ *= 75 and 80

(a)	*θ *= 65			*θ *= 70		
	
		*σ* ^2^	Time		*σ* ^2^	Time
wNRMps	2.29 × 10^-3^	5.09 × 10^-6^	14472	1.68 × 10^-4^	3.40 × 10^-8^	16140
wSSAps	2.29 × 10^-3^	5.10 × 10^-6^	16737	1.68 × 10^-4^	3.40 × 10^-8^	18555
Refined wSSA	2.29 × 10^-3^	3.39 × 10^-5^	24340	1.68 × 10^-4^	4.29 × 10^-7^	25492

**(b)**	***θ *= 75**			***θ *= 80**		
	
		***σ* ^2^**	**Time**		***σ* ^2^**	**Time**

wNRMps	8.42 × 10^-6^	1.10 × 10^-10^	15640	2.99 × 10^-7^	1.82 × 10^-13^	16260
wSSAps	8.42 × 10^-6^	1.10 × 10^-10^	18582	2.99 × 10^-7^	1.82 × 10^-13^	18960
Refined wSSA	8.43 × 10^-6^	3.58 × 10^-9^	26314	2.99 × 10^-7^	1.29 × 10^-11^	26987

### 5.2 A reaction system with *G*_1_, *G*_2 _and *G*_3 _reactions

The previous system only contains a *G*_1 _reaction and a *G*_2 _reaction. We used the following system with *G*_1_, *G*_2 _and *G*_3 _reactions to test the wNRMps and the wSSAps:(32)

In this system, a monomer *S*_1 _converts to *S*_2 _with a probability rate constant *c*_1_, while *S*_2 _is degraded with a probability rate constant *c*_2_. Meanwhile, another species *S*_3 _synthesizes *S*_1 _with a probability rate constant *c*_3 _and *S*_1 _degrades with a probability rate constant *c*_4_. In our simulations, we used the following values for the probability rate constants and the initial state:(33)

and(34)

This system is at equilibrium and the mean value of *X*_2_(*t*) is 40. We are interested in *P *(*E_R_*) = *P*_*t*≤10_(*X*_2 _→ *θ*|**x**(0)), the probability of *X*_2_(*t*) = *θ *for *t *≤ 10. We chose *θ *= 65 and 68 in our simulations. To apply the wSSAps and the wNRMps to estimate *P*(*ER*), we divide the system into three groups. The *G*_1 _group contains reaction *R*_1_; the *G*_2 _group includes reaction *R*_2_; the *G*_3 _group consists of reactions *R*_3 _and *R*_4_. When fine-tuning the parameters, we further divided *G*_3 _into a *G*_31 _group which contains reaction *R*_3 _and a *G*_32 _group which contains reaction *R*_4_. Since the system is at equilibrium and we have *a*_0_(**x**_0_) = 20, *a*_1_(**x**_0_) = 4, *a*_2_(**x**_0_) = 4, *a*_3_(**x**_0_) = 8 and *a*_4_(**x**_0_) = 4, we get , , ,  and . Therefore, we get  and the following probabilities: ,  and .

If *θ *= 65, we have *η *= 25. Using (23), we obtained *κ *= 29. Substituting *κ *into (24), we got ,  and . We then chose *α *= 0.85 and *β *= 0.80 and calculated  and  from (26) as  and . Similarly, if *θ *= 68, we got *κ *= 26, which resulted in  and . Again, selecting *α *= 0.85 and *β *= 0.80, we got  and . To test whether the wNRMps and the wSSAps are sensitive to parameters *α *and *β*, we also used another set of parameters *α *= 0.80 and *β *= 0.75.

**Figure 2:**
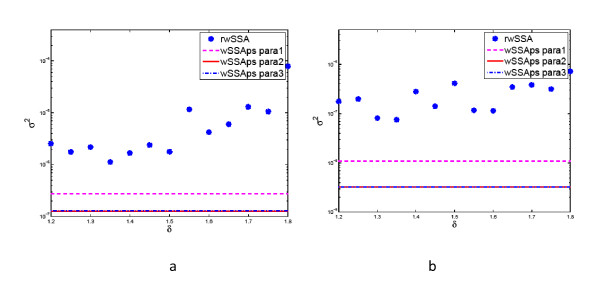
**Variance *σ*^2 ^obtained from 10^7 ^runs of the wSSAps and the refined wSSA for the system in (32) with *c*_1 _= 0.1, *c*_2 _= 0.1, *c*_3 _= 8, *c*_4 _= 0.1, *X*_1_(0) = 40, *X*_2_(0) = 40 and *X*_3_(0) = 1**. wSSAps para 1 represents the wSSAps without fine-tuning the probability of reactions in *G*_3 _group; wSSAps para 2 and 3 represent the wSSAps with fine-tuning the probability of reactions in *G*_3 _group using two sets of parameters: *α *= 0.85, *β *= 0.8 and *α *= 0.80, *β *= 0.75. Since the variance of the wSSAps does not depend on *δ *used in the refined wSSA, it appears as a horizontal line.

In order to compare the performance of the wNRMps and the wSSAps with that of the refined wSSA, we also ran simulations with the refined wSSA. In the refined wSSA, we chose the following parameters *γ*_1 _= *δ*, *γ*_2 _= 1/*δ *and *γ_m _*= 1, *m *= 3, 4 to adjust propensity functions. Since the optimal value of *α *is unknown, we ran the refined wSSA for *δ *= 1.2, 1.25, 1.3, 1.35, 1.40, 1.45, 1.50, 1.55, 1.60, 1.65, 1.70, 1.75 and 1.80 to determine the best *δ*. Figure 2 shows the variance of  obtained from the simulations with the refined wSSA and the wSSAps. Since the wNRMps yielded almost the same variance as the wSSAps, we only plotted the variance obtained from the wSSAps. It is seen that the wSSAps provides variance more than one order of magnitude lower than that provided by refined wSSA with the best *δ*. Is also observed that the wSSAps is not very sensitive to the parameters *α *and *β*, since the variance obtained with two different sets of values for *α *and *β *is almost the same.

Table 2 lists  and its variance obtained from *n *= 10^7 ^runs of the refined wSSA, the wNRMps and the wSSAps. We first ran the wNRMps and the wSSAps without fine-tuning the probability of reactions in *G*_3 _group and calculated *q_m _*using (25). We then ran the wNRMps and the wSSAps with fine-tuning the probability of reactions in *G*_3 _group and used two sets of parameters (*α *= 0.85, *β *= 0.80; *α *= 0.80, *β *= 0.75) and (26) to calculate *q_m _*for the reactions in *G*_3 _group. We also made 10^11 ^runs of the exact SSA to estimate . It is seen that the wNRMps, the wSSAps and the refined wSSA all yield the same  as the exact SSA. However, the wNRMps and the wSSAps with fine-tuning the probabilities of *G*_3 _reactions offer variance more than one order of magnitude lower than that provided by the refined wSSA. Without fine-tuning the probabilities of *G*_3 _reactions, the wNRMps and the wSSAps provided a little bit larger variance but still almost one order of magnitude lower than that provided by the refined wSSA. Table 2 also shows that the wNRMps and the wSSAps needed only 60-70% CPU time needed by the refined wSSA. Again, the CPU time of the refined wSSA in Table 2 does not include the time needed for searching for the optimal value of *δ *for each *θ*. If we include this time, the CPU time of the refined wSSA will be almost doubled.

**Table 2:** Estimated probability of the rare event  and the sample variance *σ*^2 ^as well as the CPU TIME (in s) with 10^7 ^runs of the wNRMps, the wSSAps and the refined wSSA for the system given in (32): (a) *θ *= 65 and (b) *θ *= 68

(a)		*σ* ^2^	Time
wNRMps without *G*_3 _fine-tuning	1.14 × 10^-4^	2.77 × 10^-7^	13381
wSSAps without *G*_3 _fine-tuning	1.14 × 10^-4^	2.74 × 10^-7^	17484
wNRMps with *α *= 0.85, *β *= 0.80	1.14 × 10^-4^	1.27 × 10^-7^	13504
wSSAps with *α *= 0.85, *β *= 0.80	1.14 × 10^-4^	1.28 × 10^-7^	16649
wNRMps with *α *= 0.80, *β *= 0.75	1.14 × 10^-4^	1.29 × 10^-7^	13540
wSSAps with *α *= 0.80, *β *= 0.75	1.14 × 10^-4^	1.29 × 10^-7^	17243
Refined wSSA	1.14 × 10^-4^	1.54 × 10^-6^	24499

**(b)**	****	***σ* ^2^**	**Time**

wNRMps without *G*_3 _fine-tuning	1.49 × 10^-5^	1.14 × 10^-8^	14087
wSSAps without *G*_3 _fine-tuning	1.49 × 10^-5^	1.09 × 10^-8^	17285
wNRMps with *α *= 0.85, *β *= 0.80	1.49 × 10^-5^	3.28 × 10^-9^	13920
wSSAps with *α *= 0.85, *β *= 0.80	1.49 × 10^-5^	3.29 × 10^-9^	17862
wNRMps with *α *= 0.80, *β *= 0.75	1.49 × 10^-5^	3.32 × 10^-9^	14018
wSSAps with *α *= 0.80, *β *= 0.75	1.49 × 10^-5^	3.30 × 10^-9^	17858
Refined wSSA	1.49 × 10^-5^	7.93 × 10^-8^	24739

The probability of the rare event estimated from 10^11 ^runs of exact SSA method is 1.14 × 10^-4 ^for *θ *= 65 and 1.49 × 10^-5 ^for *θ *= 68

### 5.3 Enzymatic futile cycle model

The enzymatic futile cycle model used in [22,23] consists of two instances of the elementary single-substrate enzymatic reaction described by the following six reactions:(35)

This system essentially consists of a forward-reverse pair of enzyme-substrate reactions, with the conversion of *S*_2 _into *S*_5 _catalyzed by *S*_1 _in the first three reactions and the conversion of *S*_5 _into *S*_2 _catalyzed by *S*_4 _in the last three reactions. We used the same probability rate constants and initial state as used in [22,23]:(36)

and(37)

With the above rate constants and initial state, *X*_2_(*t*) and *X*_2_(5) tend to equilibrate about their initial value 50. References [22,23] sought to estimate *P*(*E_R_*) = *P*_*t*≤100_(*X*_5 _→ *θ*|**x**(0)), the probability that *X*_5_(*t*) = *θ *for *t *≤ 100 and several values of *θ *between 25 and 40. We repeated simulations with the refined wSSA in [23] for *θ *= 25 and 40. The refined wSSA employed the following parameters *γ*_3 _= *δ*, γ_6 _= 1/*δ *and *γ_m _*= 1, *m *= 1, 2, 4, 5, and we used the best value of *δ *determined in [23]: *δ *= 0.35 for *θ *= 25 and *δ *= 0.60 for *θ *= 40.

In this system, we always have *X*_2_(*t*) + *X*_5_(*t*) = 100. So when the rare event occurs at time *t*, we have *X*_5_(*t*) = *θ *and *X*_2_(*t*) = 100 - *θ*. The rare event is therefore defined as *X*_5 _= 50 + *η *with *η *= *θ *- 50 or equivalently *X*_2 _= 50 - *η*. According to the partition rule defined in Section 4, *R*_3 _is a *G*_2 _reaction; *R*_6 _is a *G*_1 _reaction; *R*_1_, *R*_2_, *R*_4 _and *R*_5 _are *G*_3 _reactions.

We ran Gillespie's SSA 10^3 ^times and got an estimate of  as , and thus . When *θ *= 40, we have *η *= -10. Using (23) and *K_E _*= 432, we obtained *κ *= 6 Substituting *κ *into (24), we got ,  and . In this example, there always have certain reactions whose propensity functions are zero, since we always have *X*_1_(*t*) + *X*_3_(*t*) = 1 and *X*_4_(*t*) + *X*_6_(*t*) = 1. Due to this special property, we calculate the probability of each reaction as follows. The system has only 4 states in terms of *X*_3_(*t*) and *X*_6_(*t*): *X*_3_(*t*)*X*_6_(*t*) = 11, 01, 10 or 00. From the 10^3 ^runs of Gillespie's exact SSA, we estimated the probability of reactions occurring in reach state as *P*_11 _≈ 1/2, *P*_01 _= *P*_10 _≈ 1/4 and *P*_00 _≈ 0. Note that reaction *R*_6 _only occurs in states 11 and 01 and we denote its probability in these two states used in the wSSAps as  and  and its natural probability as  and . The probability  can be calculated as  and  can be approximated as  assuming *X*_2_(*t*) = 50 since the system is in equilibrium. Then, using the relationships:  and , we get  and . Reaction *R*_3 _only occurs in states 11 and 10 and its probability can be obtained similarly as  and . In a state *s *(s = 11, 01, 10 or 00), we calculate  and then calculated , *m *= 1, 2, 4 and 5, from (25). Surprisingly, , ,  and  we calculated are very close to the values used in the refined wSSA which were obtained by making 10^5 ^runs of the refined wSSA for each of seven guessed values of *γ*. In contrast, we do not need to guess the values of parameters but calculate them analytically, and all the information needed in our calculation was obtained from 10^3 ^of Gillespie's exact SSA, which incurs negligible computational overhead.

When *θ *= 25, we have *η *= -25. Using (23) and , we obtained *κ *= 3. Substituting *κ *into (24), we got ,  and . Similar to the previous calculation, we got , ,  and  and then calculated the probabilities of other reactions from (25). Again , ,  and  we obtained are very close to the values used in the refined wSSA.

**Figure 3:**
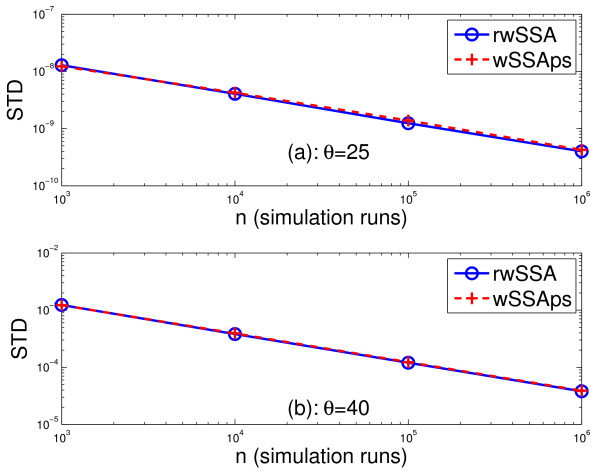
**The SD  of the estimated probability versus the number of simulation runs *n *obtained with the refined wSSA and the wSSAps for the enzymatic futile cycle model (35) with *c*_1 _= *c*_2 _= *c*_4 _= *c*_5 _= 1, *c*_3 _= *c*_6 _= 0.1, *X*_1_(0) = *X*_4_(0) = 1, *X*_2_(0) = *X*_5_(0) = 50 and *X*_3_(0) = *X*_6_(0) = 0 for *θ *= 25 and 40**.

Table 3 lists the simulation results obtained from 10^6 ^runs of the wNRMps, the wSSAps and the refined wSSA for *θ *= 40 and 25. It is seen that the estimated probability  and variance *σ*^2 ^are almost identical for all three methods, which is expected because the probability of each reaction in three methods is almost the same. This implies that all three methods may have used near optimal values for the importance sampling parameters. However, in the previous two systems, the parameters used by the refined wSSA are far away from their optimal values, because the wSSAps and the wNRMps provided much lower variance than the refined wSSA. It is also seen from Table 3 that the wSSAps used almost the same CPU time as that used by the refined wSSA and that the wNRMps used about 80% of the CPU time of the refined wSSA. Again, the CPU time of the refined wSSA does not include the time needed to find the optimal value of *δ*. Figure 3 depicts the standard deviation of the estimated probability versus the number of simulation runs *n *for the wSSAps and the refined wSSA. Since the wNRMps provides almost the same standard deviation as the wSSAps, we did not plot it in the figure. It is again seen that the wSSAps and the refined wSSA yield almost the same standard deviation for all values of *n *in this case. It was demonstrated in [24] that the dwSSA yielded comparable variance as the refined wSSA. Therefore, our parameter selection method offers similar performance to the dwSSA in this example.

**Table 3:** Estimated probability of the rare event  and the sample variance *σ*^2 ^as well as the CPU TIME (in s) with 10^6 ^runs of the wNRMps, the wSSAps and the refined wSSA for the enzyme futile cycle model (35): (a) *θ *= 25 and (b) *θ *= 40

(a)		*σ* ^2^	Time
wNRMps	1.74 × 10^-7^	1.81 × 10^-13^	4183.2
wSSAps	1.74 × 10^-7^	1.80 × 10^-13^	5316.9
Refined wSSA	1.74 × 10^-7^	1.61 × 10^-13^	5337.2

**(b)**		***σ* ^2^**	**Time**

wNRMps	4.21 × 10^-2^	1.51 × 10^-3^	3589.4
wSSAps	4.21 × 10^-2^	1.51 × 10^-3^	4388.3
Refined wSSA	4.21 × 10^-2^	1.51 × 10^-3^	4406.6

## 6 Conclusion

The wSSA and the refined wSSA are innovative variation of Gillespie's standard SSA. They provide an efficient way for estimating the probability of rare events that occur in chemical reaction systems with an extremely low probability in a given time period. The wSSA was developed based on the directed method of the SSA. In this paper, we developed an alternative wNRM for estimating the probability of the rave event. We also devised a systematic method for selecting the values of importance sampling parameters, which is absent in the wSSA and the refined wSSA.

This parameter selection method was then incorporated into the wSSA and the wNRM. Numerical examples demonstrated that comparing with the refined wSSA and the dwSSA, the wSSA and the wNRM with our parameter selection procedure could substantially reduce the variance of the estimated probability of the rare event and speed up simulation.

## Abbreviations

NRM: next reaction method; wNRM: weighted NRM; wSSA: weighted stochastic simulation algorithm.

## Competing interets

The author declares that they have no competing interests.
